# Influence of Tea Brewing Parameters on the Antioxidant Potential of Infusions and Extracts Depending on the Degree of Processing of the Leaves of *Camellia sinensis*

**DOI:** 10.3390/molecules26164773

**Published:** 2021-08-06

**Authors:** Jolanta Kowalska, Agata Marzec, Ewa Domian, Sabina Galus, Agnieszka Ciurzyńska, Rita Brzezińska, Hanna Kowalska

**Affiliations:** 1 Department of Food Engineering and Process Management, Institute of Food Sciences, Warsaw University of Life Sciences, 159c Nowoursynowska St., 02-776 Warsaw, Poland; agata_marzec@sggw.edu.pl (A.M.); ewa_domian@sggw.edu.pl (E.D.); agnieszka_ciurzynska@sggw.edu.pl (A.C.); 2 Department of Chemistry, Institute of Food Sciences, Warsaw University of Life Sciences, 159c Nowoursynowska St., 02-776 Warsaw, Poland; rita_glowacka@sggw.edu.pl

**Keywords:** tea, brewing tea, phenolic compounds, antioxidants, epigallocatechin-3-gallate

## Abstract

The polyphenol content of tea depends on the growing region, harvest date, the production process used, and the brewing parameters. In this study, research was undertaken that included an analysis of the influence of the brewing process parameters on the content of total polyphenols (Folin-Ciocalteu), epigallocatechin gallate (HPLC), and antioxidant activity (against DPPH radicals) of fresh tea shrub leaves grown from Taiwan and of teas obtained from them (oolong, green in bags, and green loose from the spring and autumn harvest). The antioxidant potential was determined in the methanol and aqueous extracts, as well as in infusions that were obtained by using water at 65 or 100 °C and infusing the tea for 5 or 10 min. The highest content of total polyphenols and epigallocatechin gallate was found in green tea extracts from the spring harvest. However, in the case of infusions, the highest content of these compounds was found in green tea in bags. Steaming at 100 °C for 10 min, turned out to be the most favourable condition for the extraction. Oolong tea, brewed at 100 °C for 5 min was characterised by the highest antioxidant activity against stable DPPH radicals.

## 1. Introduction

Tea is one of the most consumed beverages [1]. Beneficial effects on the circulatory system, improvement of heart function, prevention of atherosclerosis and obesity, anticancer, anti-inflammatory, and antibacterial properties are some of the properties of tea [2,3]. Most of them result from the presence of biologically active substances in tea leaves, mainly polyphenolic compounds, but also alkaloids (theobromine, theophylline, and caffeine) and theanine [4,5].

Tea bushes are grown mainly in Southeast Asia (China, Japan, India, Sri Lanka, Taiwan, Malaysia) but also in South America (Argentina and Brazil), Africa (Kenya), and South Africa. The amount of harvest each year in each region depends on the climate. It is believed that early harvest is better, and the tea obtained from it has better organoleptic and health-promoting qualities [6]. Moreover, the location of the plantations depending on the height above sea level has an impact on the cultivation of tea bushes. The higher it is, the better the properties of the tea. Tea is divided into three types resulting from the level of fermentation: green tea, oolong tea, and black tea [3,7].

The polyphenol content of tea depends on the growing conditions and the method of leaf processing. The dominant polyphenols in tea are catechins, which constitute about 18–36% of the dry matter of the tea leaves. They can be divided into 4 types: (−)-epigallocatechin-3-gallate (EGCG), (−)-epicatechin-3-gallate (EKG), (−)-epigallocatechin (EGC), and (−)-epicatechin (EC) [8].

The dominant compound from the group of catechins, and showing the highest biological activity, is epigallocatechin-3-gallate [9,10]. Polyphenols have a strong antioxidant effect with a scientifically proven beneficial effect on the human body, but also shape the organoleptic value of food products, e.g., taste, smell, and colour [11].

An important process taking place in tea leaves is the reaction of polyphenolic compounds (mainly catechins) catalysed by polyphenol oxidases released from the cell juice during the maceration of the raw material. This results in the condensation of catechins to tannins, the oxidation products of which significantly shape the taste, aroma features, and colour of tea. The leaves change colour, and precursors to taste and aroma are formed. Polyphenols, including those present in the leaves of tea shrubs, are pH sensitive and their properties are stable under low pH conditions [12]. Strong acids and bases, as well as light, radiation, and high temperatures, degrade poly-phenols, thus reducing their beneficial effect on the body [13].

Teas are obtained in a multistage process, which is carried out depending on the type of final product. The terminal bud and two young leaves are the most valuable, from which teas with the best tastes and aromas are obtained [5,14]. After the leaves are torn off, they are left to wilt and then twisted or chopped [15,16]. The exception is white tea, which is dried after harvesting. The duration of each step depends on the processing method. Drying is carried out in natural conditions (in the sun or in solar dryers) and manually or mechanically. Currently, twisting machines are most often used, which crush and roll the leaves to extract the cell juice from them, which determines the final taste of the tea. The next step is the fermentation (oxidation) process. This stage is for black teas. Oolong tea is produced by a short fermentation called semi-fermentation [5]. In contact with oxygen, catechins contained in the leaves oxidise, and the leaves darken, which confirms the initiation of the fermentation process [17]. The first sifted leaves are referred to as “first fine” and are of the highest quality due to the high content of young, small leaves and buds. As a result of oxidation, the tannins are reduced (the taste is milder), caffeine is activated, and the activation or formation of new essential oils influences the tea aroma. However, in the production of green and yellow teas, this stage is replaced by treating the leaves with high temperature. In Japan, the leaves are steamed at about 100 °C, while in China, panning is used at 300–350 °C. The technique is designed to deactivate enzymes, which has a protective effect on polyphenols and chlorophyll, which does not break down and maintains a green colour [18]. Then, the tea leaves are dried at the temperature of 85–88 °C for about 20 min, during which the water content is reduced to about 3%, the value decisive for the stability of the tea during storage [19].

According to Shannon et al. [20], water temperature and tea infusion time have a significant impact on the extraction efficiency of compounds such as polyphenols and methylxanthines (mainly caffeine, theophylline, and theobromine). At home, the brewing temperature is usually in the range of 65–95 °C, while green and white tea is usually brewed at a lower temperature than black tea. As emphasised by Shannon et al. [20], some studies show that a higher polyphenol content in *Camellia sinensis* teas can be achieved with longer brewing times (>10 min), but this mainly applies to the extraction of compounds in industrial applications, as astringency and bitterness increase over time, affecting the organoleptic properties of the drink. Infusing loose leaves compared to packaged or powdered tea can affect the extraction rate and compounds content [21].

Teas are used as an additive to other food products, including for the production of ice cream, bread, bars, and even for animal feed. Most of all, however, they are consumed in the form of a drink as a valuable source of natural antioxidants that have a beneficial effect on the human body. Therefore, it is necessary to know the influence of temperature and brewing time on the behaviour of these valuable compounds. Most studies compare the antioxidant properties of commercial teas from different growing regions.

In this study, research was undertaken including the analysis of the antioxidant potential expressed in the total content of polyphenols, epigallocatechin gallate content, and antioxidant activity in fresh tea tree leaves and teas obtained from them, as well as the influence of the brewing process parameters on the content of the analysed compounds in the research material from one region on Taiwan.

## 2. Results

Preliminary studies showed no statistically significant differences in the polyphenol content between infusions brewed for 3 and 5 min of brewing at the temperature of 85 °C, similarly to other temperature values. Therefore, for further research, brewing for 5 and 10 min at 65 and 100 °C was used. In addition, methanol and water extracts were prepared to determine the antioxidant potential.

### 2.1. Dry Matter Content

The dry matter content in the fresh leaves was 98.4%. All analysed teas were characterised by a similar dry matter content at the level of over 99%. The water content in teas should not exceed 3%.

### 2.2. Total Polyphenol Content

#### 2.2.1. Methanol and Water Extracts

**Figure 1:**
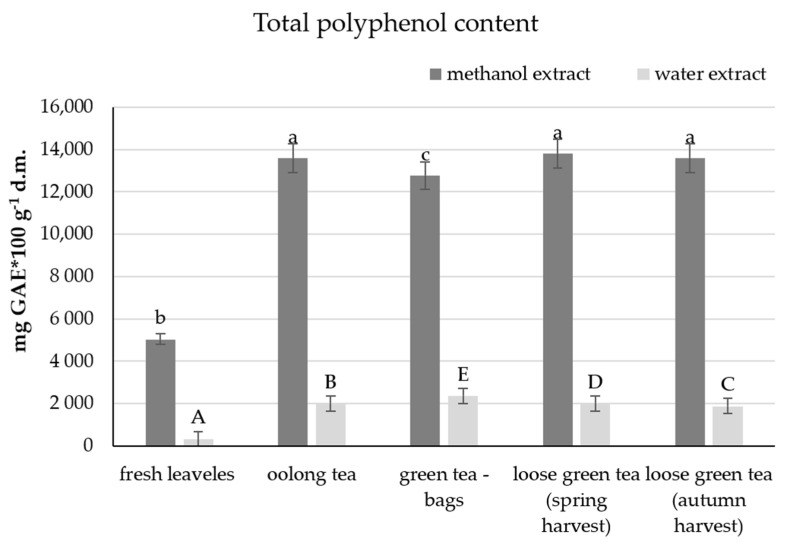
Total polyphenol content in the leaves of the tea plant and various types of tea. The same letter a, b, c indicates no statistically significant differences between the mathanol extracts, and A, B, C—between the water extracts of the analyzed teas.

The content of polyphenols extracted with 80% methanol in fresh tea leaves was 5032 mg gallic acid per 100 g dry matter (Figure 1). It was a value over 2.5 times lower than the values determined in the analysed teas. The lowest total polyphenols (approx. 12,770 mg of gallic acid in 100 g of dry matter) were determined in green tea bags. The analysis of green teas in bulk showed a slightly higher content of polyphenols in tea from the spring harvest compared to the autumn harvest.

The second variant involved the determination of total polyphenols extracted with water. The obtained results were significantly lower compared to the methanol extracts. The smallest amount of polyphenols was determined in fresh tea leaves (325 mg gallic sq in 100 g dry matter), and it was a value approximately seven times lower than the results obtained for teas. In teas, the least polyphenols were determined in green tea, in bulk, from the autumn harvest (1876 mg GAE per 100 g d.m.), while the highest in oolong tea (2356 mg GAE w 100 g d.m.).

The dominant catechin among the polyphenols in tea, characterised by the highest antioxidant activity, is epigallocatechin gallate. The content of this compound was analysed in the water and methanol extracts.

**Figure 2:**
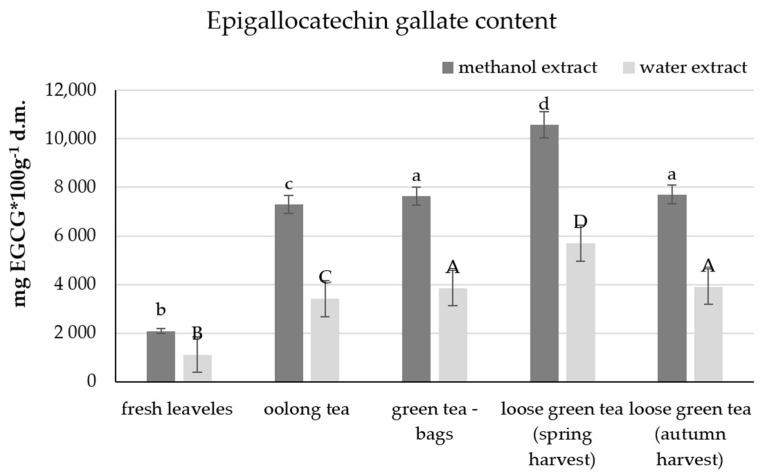
Epigallocatechin gallate content in the leaves of the tea plant and various types of tea. The same letter a, b, c indicates no statistically significant differences between the mathanol extracts, and A, B, C—between the water extracts of the analyzed teas.

As in the analysis of total polyphenols, the least epigallocatechin gallate was found in fresh leaves (2098 mg EGCG per 100 g d.m. in methanol extract and 1121 mg per 100 g d.m. in water extract) (Figure 2).

The determined content at the level of 10,578 mg of EGCG in 100 g of d.m. in the methanol extract and 5698 mg in the aqueous extract, which was respectively more than 2860 mg EGCG and 1840 mg higher compared to the same type of tea but from the autumn harvest. Similar content of epigallocatechin gallate was determined in oolong tea and green tea in tea bags. The highest amount of catechin was found in leaf green tea (loose) from the spring harvest.

**Figure 3:**
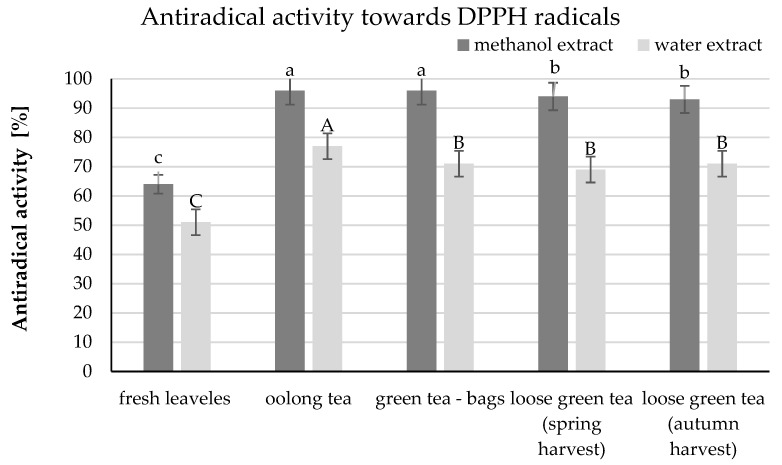
Antiradical activity towards DPPH radicals in the leaves of the tea plant and various types of tea. The same letter a, b, c indicates no statistically significant differences between the mathanol extracts, and A, B, C—between the water extracts of the analyzed teas.

An important indicator of the antioxidant potential is the ability of polyphenols to scavenge free radicals, and thus the antiradical activity. In the present study, it was expressed as antiradical activity against stable DPPH radicals. All methanol extracts showed an activity of over 90% and were found in three homogeneous groups (Figure 3). The oolong and green tea extracts in bags (96%) showed the highest activity. The lowest activity was observed in samples of green tea in bulk from the autumn harvest (93%). The aqueous extracts were more varied. The lowest activity was found in fresh leaf extracts (61%), while the highest activity was that of oolong tea (77%). Green tea extracts in bags and green tea in bulk from the autumn harvest showed lower activity by 6 percentage points.

A strong positive correlation was demonstrated between the total content of polyphenols and the antioxidant activity of DPPH radicals, which was 0.9847 (Pearson’s correlation). A slightly lower correlation at the level of 0.8829 was shown between the epigallocatechin gallate content and the ability of phenolic compounds to scavenge DPPH free radicals. The obtained dependencies show that epigallocatechin gallate significantly influences the antioxidant activity of the teas tested.

#### 2.2.2. The Infusions

The next stage of the research was to assess the antioxidant potential of the infusions of the studied leaves and teas. In accordance with the information included in the research methodology (Section 4.2), the infusions were prepared using water at a temperature of 65 and 100 °C and brewing for 5 and 10 min (conditions corresponding to those used by consumers at home).

**Figure 4:**
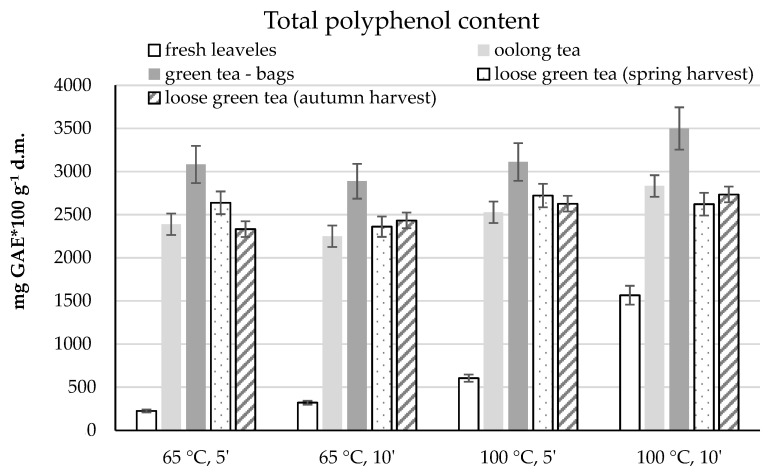
The content of total polyphenols in water infusions of tea shrub leaves and various types of teas, depending on the brewing parameters.

The lowest total polyphenol content was found in infusions prepared from fresh leaves. In samples prepared by steaming in water at 65 °C for 5 min, 225 mg of GAE in 100 g of d.m. was determined (Figure 4). Brewing at this temperature for 5 min showed the highest amount of total polyphenols in teabag green teas (3083 mg), which was about 14–24% higher than the other analysed teas. In most of the analysed samples, extending the infusion time to 10 min resulted in a reduction in the content of the tested compounds.

The use of water at 100 °C increased the extraction efficiency of polyphenolic compounds to about 605 and 1566 mg GAE in 100 g d.m. after 5 and 10 min of brewing, respectively. The highest content of total polyphenols was found in green tea infusions in bags, regardless of the parameters used. The 10 min infusion in water at a temperature of 100 °C resulted in the highest content of polyphenolic compounds (about 3500 mg GAE in 100 g d.m.) (Figure 4).

About 400 mg less of the analysed compounds were determined in the infusions of this tea infused for 5 min at both temperatures.

In other tea infusions, the influence of the brewing parameters used was not unequivocal. Oolong tea showed an increase in the content of polyphenols extracted into the infusion with increasing temperature and increasing brewing time, except for the results obtained after 10 min at 65 °C. Green teas, in bulk, from different harvest periods were characterised by an ambiguous influence of the parameters used. After 5 min of brewing in both temperature variants, the higher content of polyphenols was determined in teas from the spring harvest. However, after 10 min of brewing, more polyphenols were determined in the samples from the autumn harvest. Statistical analysis showed significant differences between these teas, which indicates the effect of harvest time on the content of polyphenols in teas (Table 1).

**Figure 5:**
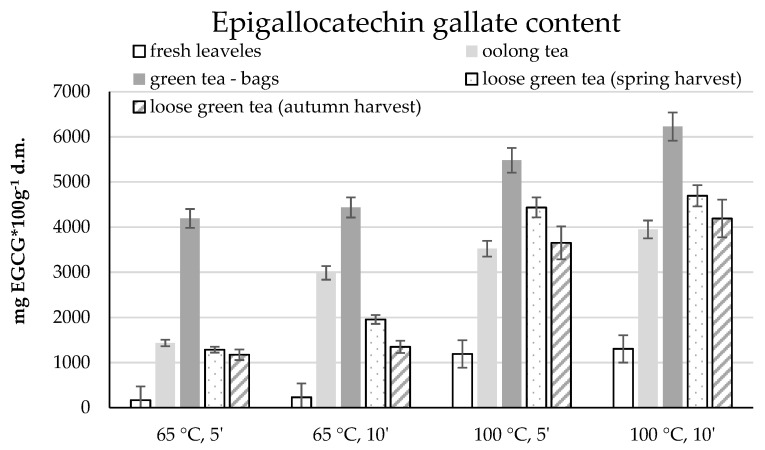
Epigallocatechin gallate content in water infusions of tea shrub leaves and various types of teas depending on the brewing parameters.

The dominant catechin in teas, epigallocatechin gallate (EGCG), was also determined in infusions. As in the case of the analysis of the total polyphenol content, green tea in bags stood out. In each of the brewing variants, the most epigallocatechin gallate was determined in the infusion of this tea. Moreover, the influence of both time and temperature on the degree of extraction of the test compound was shown. The lowest level of epigallocatechin gallate was determined in infusions brewed in water at 65 °C for 5 min, i.e., 4191 mg EGCG per 100 g d.m (Figure 5).

About 240 mg more EGCG was determined in the infusion after 10 min of infusion. About 5478 and 6227 mg of EGCG, respectively, were infusions obtained with 100 °C water after 5 and 10 min. Similar dependencies were obtained in the remaining infusions. The obtained results showed a higher content of epigallocatechin gallate in loose tea from the spring harvest compared to tea from the autumn harvest. Apart from infusions steamed for 5 min at 65 °C (the difference in epigallocatechin gallate content was approx. 110 mg), in the other variants, the content of the determined compound in infusions of spring teas was from about 500 to over 780 mg higher (Figure 5).

Oolong tea is also noteworthy. Brewing at 65 °C resulted in the extraction of approximately 1435 and 2989 mg of EGCG after 5 and 10 min, respectively, and these were higher than those determined for bulk green teas. On the other hand, brewing at 100 °C resulted in a higher extraction of epigallocatechin gallate, especially in green tea in bulk from spring harvest, by more than 900 and 740 mg of EGCG after 5 and 10 min, respectively.

The statistical analysis confirmed the significant influence of both temperature and brewing time on the epigallocatechin gallate content in the examined leaves and teas (Table 2). The influence of the harvest date on the content of this compound was also shown.

**Figure 6:**
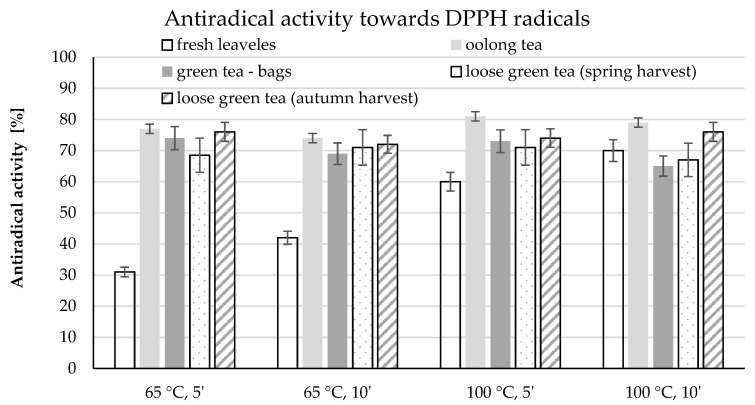
Antiradical activity towards DPPH radicals in water infusions of tea shrub leaves and various types of teas depending on the brewing parameters.

Although the highest content of total polyphenols and epigallocatechin gallate was determined in green tea infusions in bags, these infusions were not characterised by the highest radical scavenging capacity. The highest antiradical activity was demonstrated in oolong tea infusion in each of the brewing variants (Figure 6).

The statistical analysis confirmed the influence of the brewing parameters on the ability of the polyphenols contained in the infusions to scavenge DPPH water radicals (Table 3). There was no unequivocal relationship between the parameters used and the antiradical activity of the infusions. The infusion of fresh leaves showed a similar tendency as in the case of other determinations, i.e., an increase in temperature and an extension of the infusion time resulted in higher antiradical activity. The highest activity was shown by oolong tea in the form of an infusion obtained at a temperature of 105 °C for 5 min (81%) (Figure 6).

Oolong and green teas in bags showed lower antiradical activity of infusions prepared at the same temperature but for 10 min. Oolong tea infusion at 65 °C decreased from 77% to 74% after 10 min of brewing. A similar relationship was shown in infusions obtained at 100 °C (81%, 79%, respectively). The content of total polyphenolic compounds and epigallocatechin gallate in green teas in bulk from both crops was not reflected in the determined antiradical activity (Figure 6, Table 3). The antiradical activity of the infusions ranged from 65% for the infusion of green tea in bags to 81% for the infusion of oolong tea brewed at 100 °C for 10 and 5 min, respectively.

The analysis of the correlation between the content of total polyphenols and the antioxidant activity showed that with increasing temperature and extending the extraction time in individual variants, the relationship between the analysed values decreased. Low and negative correlation was demonstrated between the content of polyphenols and the antiradical activity of infusions prepared at 100 °C for 10 min. In the remaining variants, these dependencies were highly and positively correlated. Similar relationships were demonstrated by comparing epigallocatechin gallate content and antioxidant activity. In this case, the force of the impact was much lower (Table 3).

## 3. Discussion

Green tea is one of the most consumed beverages in the world [3]. It is related to the properties of this drink, both organoleptic and pro-health. As shown in their research by Kompes et al. [22] and Jakubczyk et al. [23] the health-promoting effect of green tea is mainly attributed to the presence of polyphenols, especially flavanols and flavonols, which constitute about 18–30% of the dry matter content of fresh leaves. Literature data show that green tea is dominated by catechin (−)-epigallocatechin-3-gallate (EGCG) which are attributed with many beneficial pro-health properties [17]. In the present study, epigallocatechin gallate accounted for over 53% (in the case of oolong tea) to over 76% (in the case of spring-harvest green tea) of total polyphenols. This is in line with the results obtained by Bae et al. [24] who showed that EGCG is the dominant catechin in green tea, accounting for 50–80% of all polyphenols. It is also believed to be a major contributor to the various health benefits of green tea [8,25].

As demonstrated by Li et al. [4], the content of antioxidant compounds in tea depends on many factors and decreases with the age of the plant. The processes to which the fresh leaves of the tea tree are subjected, during which processes occur mainly within phenolic compounds, shaping the organoleptic and health values, have a significant impact [26]. The main chemical changes of tea polyphenols during processing are oxidation, hydrolysis, polymerization, and transformation. In this study, the highest content of total polyphenols was found in green tea loose from spring harvest. Green tea is not subjected to fermentation, during which numerous processes take place, including oxidation, resulting in greater losses of polyphenols. However, as shown by the results of this study, the high content of total polyphenols was determined in oolong-semi-fermented tea too. These values were approximately three times higher than those found in fresh leaves. Similar conclusions were reached in their research by Chacko et al. [27] and Lee et al. [17]. The content of tea polyphenols is one of the most important indicators affecting the quality of tea. In the process of obtaining green tea, oxidase and other enzymes are inactivated by exposing the leaves to high temperature [17]. In addition, the stage of twisting the leaves (destroying cells and releasing juice) and fermentation are important, during which the access of oxygen increases and oxidation is enhanced mainly by polyphenol oxidase and peroxidase [28]. The result is the formation of polyphenols with a higher molecular weight, mainly due to the oxidation of catechins to theaflavins. Moreover, the longer the fermentation time, the greater the degradation of the polyphenols as a result of greater and longer exposure to enzymes and oxygen. Therefore, it is considered that black teas contain the least polyphenolic compounds, slightly more of them are present in oolong (semi-fermented) teas, and the most in green, white or yellow teas [10]. This study did not confirm the literature data. Oolong tea contained a similar polyphenol content as green tea, as mentioned earlier. It was probably due to the content of theaflavins showing strong antioxidant properties, and being formed, among others, during the fermentation process.

Lee et al. [17] showed that the action of high temperature during the production of green teas has a significant, positive impact on the content of polyphenolic compounds. The results of this research confirm the conclusions of the authors mentioned. The total content of polyphenols-as mentioned earlier-was comparable in all the teas tested and over 2.5 times higher than in fresh leaves. The authors showed also that the content of EGCG and ECG increased about two-fold after “roasting” compared to the content of these compounds in fresh leaves. In the current studies, the determined content of epigallocatechin gallate was about 4 to more than 5 times higher than in fresh leaves. Moreover, the content of the determined compound was about 3000 mg higher in green loose tea from the spring harvest compared to the other analysed teas. These teas are obtained from delicate, selected young leaves, which is confirmed by the research of Li et al. [4], who showed that these raw materials contain the highest amount of epigallocatechin gallate. These results also indicate the effect of harvest time on EGCG content. The difference was over 27% in favour of spring-harvested teas. Moreover, a lower content of epigallocatechin gallate was found in green teas obtained from the autumn harvest (about 2500 mg) compared to tea from the spring harvest. Such a relationship has been confirmed in their research by, among others, Blum-Silva et al. [29] and Chu et al. [8]. Noteworthy, however, is the fact that a similar content of epigallocatechin gallate in oolong (semi-fermented) tea and green tea, in bags and loose, from the autumn harvest was marked. It is probably due to the presence of other forms of antioxidant compounds that are present in teas and are produced from monomeric catechins.

Although the dominant epigallocatechin gallate (EGCG) in green tea has similar antioxidant power to the main polyphenolic compound in black tea (theaflavin), the difference in antioxidant capacity between the two teas is based on the concentration of polyphenols, of which there are about twice more in green tea, according to Kim and Kim [6]. Changes under the influence of external factors may differ for individual groups of polyphenols. The present tests showed differences in the epigallocatechin gallate content between oolong and green tea, but no differences in total polyphenol content were found. The obtained results are similar to the studies carried out by Shannon et al. [20], who indicated a higher content of total polyphenols in green tea compared to black tea, but these differences amounted to about 50 mg. However, the authors showed differences at the level of individual groups of polyphenolic compounds.

Tea available for sale comes in three forms; in the form of leaves (loose), in bags, or in the form of a powder [23]. Each of these forms differs from each other in both the selection of the raw material and the degree of fragmentation. Leaf tea (loose) is made of good quality leaves, not broken, properly twisted. Tea in bags is finer, often the leaves that remain after selecting the raw material for the leaf teas.

In addition to the properties shaped on plantations depending on the cultivation region, altitude, harvest time, an important factor influencing the organoleptic and health benefits of teas are the brewing parameters [14]. On the packaging, manufacturers indicate the recommended time and temperature of tea brewing, which, as shown by numerous studies, including current ones, have an impact on the properties of infusions. Polyphenolic compounds, in addition to their health-promoting properties, affect taste, aroma, and colour.

The analysis of the antioxidant potential of infusions showed the highest content of total polyphenols and epigallocatechin gallate in green tea in bags. In all brewing variants, these infusions showed the highest content of the compounds determined, which is consistent with the results of the research conducted by Jakubczyk et al. [23]. The green tea in the bags was finer, which facilitated and accelerated the extraction of polyphenolic compounds. The authors showed that the increase in the brewing temperature resulted in greater extraction of polyphenolic compounds. However, these relationships were ambiguous and differed for the analysed types of tea. In this study, the brewing parameters also had an impact, especially for green tea infusions in tea bags. In the remaining analysed teas, no unequivocal influence of the brewing parameters on the content of the determined compounds was demonstrated. Most of the samples showed a higher content of total polyphenols in infusions prepared at a temperature of 100 °C. The brewing time had a smaller and inconclusive effect on the content of the determined compounds, although the statistical inference showed significant differences. Komes et al. [22] in their research, the highest total polyphenols were determined in green tea bags and Matcha (powdered) tea, brewed at 100 °C. The authors [22] also showed that EGCG is the dominant catechin in teas, and they marked it the most in green tea in bags and the least in leaf tea. These results are in line with those obtained in this study, as described at the beginning of this chapter. Kim and Kim [6], in their research, showed that the pH of early teas (harvest time) was lower than that of teas harvested in the following months. Low pH value affects the behaviour of polyphenolic compounds. The authors confirmed that teas from earlier harvest were characterised by a higher content of polyphenols, also after infusion, which was justified by the protective effect of low pH against oxidative degradation caused by extraction and high temperature during the preparation of infusions. In these studies, the overall content of polyphenols did not unequivocally confirm the studies by Kim and Kim [6] because the determined content of total polyphenols in green teas in bulk from both harvest periods was at a similar level. On the other hand, a statistically significant difference was found in the content of epigallocatechin gallate, which was determined by over 2800 mg more in tea loose from the spring harvest. It was especially noticeable with increasing temperature and increasing brewing time. The analysis of infusions prepared at 65 C also showed a high content of epigallocatechin gallate in oolong tea. The obtained values were higher compared to loose green teas. This may be due to the increase in gallic acid content after the fermentation process, as demonstrated in the studies by Kim and Kim [6]. Gallic acid has a strong antioxidant effect, which also affects the overall activity of teas. On the other hand, Kim and Kim [6] showed in their studies that during the fermentation of the four main flavan-3-ols (EGCG, EGC, EC, and EKG) present in tea, they are transformed into dimers and/or polymers, thus their content is degraded. The authors also confirmed that more galloflavanols are degraded, which are less stable after oxidation because they are more reactive to oxygen. This may explain the lower content of epigallocatechin gallate in the analysed oolong (semi-fermented) tea compared to the tested green teas, which are exposed to high temperature, which results in inactivation of enzymes, including polyphenol oxidase.

The analysed infusions showed high antiradical activity at the level of over 60 to 81%. These results are similar to those obtained by Jakubczyk et al. [23]. Water infusions infused for 5 min at 100 °C were characterised by the highest activity. Higher temperature results in greater release of biologically active compounds. On the other hand, exposure for too long to high temperature may lead to the degradation of polyphenolic compounds, thus reducing their antioxidant potential. Fujioka et al. [30] showed that ground teas do not require long brewing, because from this form, biologically active compounds are faster and easier to extract, which is also confirmed by the results obtained in this study.

## 4. Materials and Methods

### 4.1. Materials

**Figure 7:**
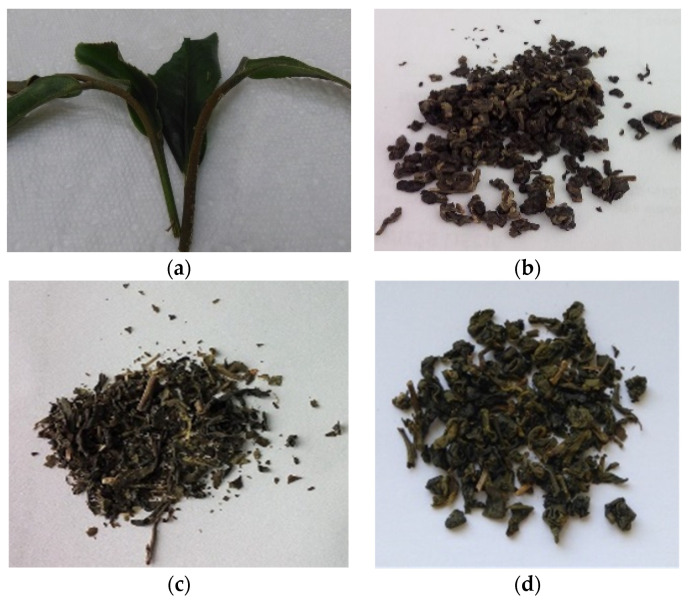
Photos of the research material: (**a**) fresh tea leaves, (**b**) loose green tea, (**c**) green tea in bags, (**d**) oolong tea (own study).

The research material consisted of fresh leaves of the tea tree *Camellia sinensis* and 4 teas obtained from the same plantation in Taiwan in the JiaYi region. Plantations are located at an altitude of about 1500 m above sea level. Fresh leaves from the spring harvest (Figure 7a) and teas: green leaves from the spring and autumn harvest (the teas did not differ visually, one photo is provided) (Figure 7b), green in bags from the spring harvest (Figure 7c) were used for the study as well as oolong in bags from the spring harvest (Figure 7d). The fresh tea leaves were harvested for research by Mr. Lai Hsin-Yong, a doctor from Taiwan, and a tea connoisseur (tea tippers). The leaves were then tightly packed in tea packaging and delivered to the test site (WULS, Warsaw, Poland). The research was started 48 h after the harvest. Green teas (sold loosely or in bags) and oolong, obtained from leaves harvested from the same plantation in the spring, were also used in the research. In addition, the research material was also loose green tea obtained from the autumn harvest, from the same plantation. According to the information obtained from the grower, the tea leaves are harvested by hand and then spread out on the ground for 3–4 days to be pre-dried in the sun.

Then the stalks are removed and the leaves are manually rolled, depending on the type of tea, along or across the leaf. After this stage, the tea leaves are dried in electric ovens at a temperature of 50–80 °C. The longer this stage takes, the darker the colour of the tea becomes. The finished tea is sorted and packed in airtight packages. Oolong tea is fermented briefly after rolling, then dried and packaged. Uncrushed leaves are selected for loose teas, while the remaining leaves are dispensed into teabags.

The planning of the experiment was based on literature data, preliminary research, and consumer knowledge. It was decided that the tests would be carried out in methanol extracts (to determine the total content of assayed phenolic compounds present in the test material) and aqueous extracts in order to compare the amounts of extracted compounds after using the extractant used by consumers. The extraction conditions were the same. This part of the experiment was to determine the content of selected phenolic compounds and to indicate the differences in their extraction depending on the extractant used. In addition, in the next part of the experiment, infusions were prepared in accordance with the recommendations of tea producers, literature data, and information on internet forums devoted to brewing tea. The infusions were prepared using different parameters of water temperature and extraction time/brewing than those used in the preparation of extracts for chemical determinations.

### 4.2. Brewing Parameters-Preparation of Infusions

In the first stage, preliminary tests were carried out to determine the brewing parameters. The process was carried out at the temperature of 65, 85, and 100 °C for 3, 5, and 10 min (using the literature data and also the recommendations of tea producers).

### 4.3. Preparation of Water and Methanolic Tea Extracts

Methanol extract and water extract were prepared for the research. The choice of the solvent was based on the literature data, as well as on the results of preliminary tests, during which 70 and 80% acetone and methanol were also used. The obtained results indicated that the most effective extractant is 80% methanol, which was used in this study. In addition, water extracts were also prepared as a result of the method of preparing tea (beverage) by consumers by pouring water over tea. The analyses carried out for water extracts were aimed at determining the content of polyphenolic compounds that are extracted during the preparation of the drink at home.

In order to prepare methanol and water tea extracts, approx. 1 g of a tea was weighed into glass bottles and poured with 50 mL of 80% methanol or distilled water (at the ambient temperature). The extracts were then placed for 30 min in a water bath (JWE 357, ELPIN+, Warsaw, Poland) at 85 °C with shaking (225 cycles/minute at 2 amplitude). After cooling, the extracts were filtered through a filter paper. To prepare water infusions, about 1 g of a given tea was weighed into glass bottles, then poured with boiled water at a temperature of 65 or 100 °C and placed in a water bath (JWE 357, ELPIN+, Warsaw, Poland) at the same temperature as the water used to brew the tea. The samples were left in the bath for 5 or 10 min (conditions consistent with the consumption of tea by consumers). After cooling, the samples were filtered through a filter paper. Methanol and water extracts as well as water infusions were prepared in 3 replications, and each of the prepared extracts and infusions was analysed in five parallel replications.

### 4.4. Analytical Methods

#### 4.4.1. Determination of the Dry Matter Content

The dry matter content [31] was determined using the drying method. About 1 g of the fresh leaves and each tea were weighed into cups and dried at 105 °C for 3 h in the laboratory chamber oven type SUP-65 WG manufacturer WAMED. The dry matter content of the samples was calculated from the difference of weights before and after drying.

#### 4.4.2. Determination of Total Polyphenols by the Folin-Ciocalteu Method

**Figure 8:**
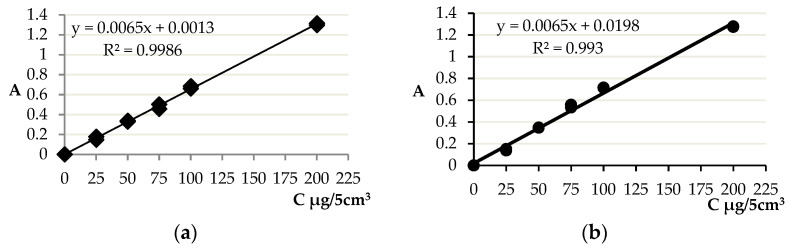
Standard curves of gallic acid in (**a**) methanol and (**b**) in water.

Total polyphenol content was determined using the Folin-Ciocalteu method [32]. In the first stage, an aqueous and methanolic gallic acid solution was prepared for the preparation of a standard curve (Figure 8). Working standard solutions of gallic acid at different concentration levels (0, 15, 25, 50, 75, and 100 mg/L) were prepared by dilution of the stock solution and used to build a calibration curves.

For the determination, 300 µL of filtered infusion of fresh leaves or tea or 75 µL of methanol or water extract were taken, which were made up to 300 µL with extraction solution. Then 4.15 mL of deionised water, 500 µL of 20% sodium carbonate solution, and 50 µL of Folin-Ciocalteu reagent were added. The reagents were mixed and allowed to stand for 20 min. The absorbance was measured at a wavelength of 700 nm on a SHIMADZU UV-1201V (Japan) spectrophotometer. Total polyphenol content was calculated on the basis of a standard curve (prepared for each variant) and expressed as gallic acid equivalent per 100 g of product dry matter.

#### 4.4.3. Analysis of Epigallocatechin Gallate Content in Tea Infusions as Well as Methanol and Water Extracts by HPLC Method

**Figure 9:**
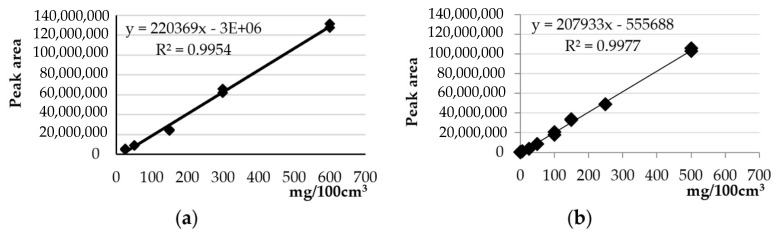
Standard curves of epigallocatechin gallate in (**a**) methanol and (**b**) in water.

The analysis of epigallocatechin gallate content in tea infusions, as well as methanol and water extracts, was performed using the HPLC method [22] with consideration of own modification. In the first step, standard curves of the epigallocatechin gallate standard (PHR 1333 SIGMA-ALDRICH), dissolved in methanol or in water (10 µL of standard dissolved in 10 mL of solvent), were prepared (Figure 9). Prepared infusions and extracts were centrifuged for 3 min at 12,000 rpm with a Centrifuge MPW-210 (MPW, Warsaw, Poland) and filtered with a syringe filter (nylon filter, pore size 45 μm). The injection volume was 10 μL for the samples tested and for the standard.

In order to prepare the mobile phase, acetic acid *p*.a. was mixed with distilled water to obtain a 3% solution. This solution was then filtered using a Supelco 58,061 Mobile Phase Filtration Kit with 0.45 μm pore nylon filters (Membranes Nylon 66 0.45 um PK/50, Supelco, Bellefonte, PA, USA). A Laboport N810 FT.18 vacuum pump (KNF Neuberger) was used to create a vacuum. The acetic acid solution prepared in this way was poured into a bottle and placed in an ultrasonic bath for 30 min.

The analysis was performed using a Kinetex 5μ C18 100 A column (Phenomenex, Torrance, CA, USA). The mobile phase was HPLC grade methanol (phase B) and 3% acetic acid (phase A) at a phase flow of 0.6 mL/min. A gradient elution was applied starting at 2% B, yielding 15% B in 25 min, 35% B between 40 and 55 min, and ending with 90% B in 55 min. Then it was reverted to the initial conditions (2% B) and held until 60 min. Compound detection was carried out with a Shimadzu SPD-M10A VP (Kyoto, Japan) diode detector with a data range of 190–800 nm. Chromatograms were recorded at 275 nm. Identification of epigallocatechin gallate was carried out by comparing the retention times of compounds contained in infusions, extracts, and standard solutions and with the help of literature data. The concentration of epigallocatechin gallate in the samples was calculated on the basis of the obtained area under their peaks and on the basis of standard curves. Final results were expressed as mg epigallocatechin gallate per 100 g dry matter content of the product.

#### 4.4.4. Determining the Ability of Extracts to Inactivate Stable DPPH Radicals

The ability of extracts to inactivate stable DPPH radicals was determined according to Drużyńska et al. [33] and Bienia et al. [34]. The DPPH solution was prepared by weighing out 19.7 mg of radicals, transferring them to a 100 cm^3^ flask, and filling up to the mark with methanol. Three types of samples were prepared for the measurement of absorbance. Specific sample, containing 4 cm^3^ of methanol or water extract and 1 cm^3^ of DPPH radical solution. The blank contained 4 cm^3^ of methanol or water extract and 1 cm^3^ of methanol. Control sample, which contained 4 cm^3^ of methanol or water (the extraction solution) and 1 cm^3^ of DPPH radicals. The samples were mixed and left to stand for 30 min, then the absorbance was measured on the NOVASPEC II Pharmacia (Sweden) spectrophotometer (zeroing the apparatus for the blank test) at a wavelength of 517 nm [35]. The percentage activity for scavenging DPPH stable radicals was calculated from the formula:

Act. = [(Ak − As)/Ak] × 100(1)

where:

Act.—antioxidant activity [%],
Ak—the absorbance of the control sample,
As—absorption of the specific sample.

### 4.5. Statistical Analysis

Statistical analysis of the results was performed using the analysis of variance, based on the ANOVA summary table (StatSoft-Statistica 13.0). Tests were carried out to check the assumption of homogeneity of univariate and multivariate variance (adopted significance level *p* = 0.05). Significant differences between measures were determined using Dukan’s multiple tests. The *p*-values below 0.05 were considered statistically significant.

## 5. Conclusions

Tea is a good source of polyphenolic compounds, mainly epigallocatechin gallate, with high antioxidant activity. The processes that the tea leaves are subjected to have an important, positive role in shaping the antioxidant potential. The influence of brewing parameters on the efficiency of extraction of polyphenolic compounds was demonstrated. In most of the analysed samples, the higher the temperature and the longer brewing time, the more compounds were determined in the analysed infusions.

The degree of crushing of the tea is important for the efficiency of extraction when preparing infusions. The best properties were characterised by green tea in bags, which is obtained from broken, crushed leaves and separated after sorting the teas in bulk.

The tested teas differed in the degree of processing, and within one type of tea, the influence of the harvest date, as well as the degree of grinding, was demonstrated. All teas were characterised by a high content of total polyphenols, epigallocatechin gallate, and high anti-radical activity. Green teas in bulk and oolong tea (semi-fermented tea classified as black teas) showed a similar content of polyphenols, and these values were several times higher than the content of these compounds in fresh leaves.

In summary, the tea is a very good source of polyphenols, and it has a high antioxidant potential, especially after brewing for 10 min at 100 °C.

## Figures and Tables

**Table 1:** Statistical analysis of the influence of brewing parameters on the total polyphenol content.

**Product**	**Brewing Parameters**	**65 °C**		**100 °C**	
		**5 min**	**10 min**	**5 min**	**10 min**
	Fresh leaves	D, a’,	c, a’,	E, E’,	d, F’,
	Oolong tea	B, b’,	e, b’, c’	A, A’	ab, D’
	Green tea–bags	C, g’	b, h’	C, G’	i, H’
	Green tea–loose–spring harvest	A, f’	f, d’	F, C’, D’	h, A’, B’
	Green tea–loose–autumn harvest	B, c’, d’	g, e’	A, A’, B’, C’	a, C’, D’

The same letters in columns mean no statistically significant differences at the confidence level of *p* ≤ 0.05. A,B,C—effect of temperature 65 °C and 100 °C during 5 min; a,b,c—effect of temperature 65 °C and 100 °C during 10 min; a’,b’,c’—effect of time 5 and 10 min at 100 °C; A’,B’,C’—effect of time 5 and 10 min at 65 °C.

**Table 2:** Statistical analysis of the influence of brewing parameters on the epigallocatechin gallate content.

**Product**	**Brewing Parameters**	**65 °C**		**100 °C**	
		**5 min**	**10 min**	**5 min**	**10 min**
	Fresh leaves	A, a’	b, b’	C, A’	a, B’
	Oolong tea	E, f’	d, I’	F, C’	e, E’
	Green tea–bags	H, j’	g, k’	J, l’	i, J’
	Green tea–loose–spring harvest	D, d’	c, g’	I, G’	h, H’
	Green tea–loose–autumn harvest	B, c’	a, e’	G, D’	f, F’

The same letters’ mean no statistically significant differences at the confidence level of *p* ≤ 0.05. A,B,C—effect of temperature 65 °C and 100 °C during 5 min; a,b,c—effect of temperature 65 °C and 100 °C during 10 min; a’,b’,c’—effect of time 5 and 10 min at 100 °C; A’,B’,C’—effect of time 5 and 10 min at 65 °C

**Table 3:** Statistical analysis of the influence of brewing parameters on the antiradical activity.

**Product**	**Brewing Parameters**	**65 °C**		**100 °C**	
		**5 min**	**10 min**	**5 min**	**10 min**
	Fresh leaves	B, e’	d, f’	C, C’	a, A’
	Oolong tea	F, g’	f, a’	G, H’	h, G’
	Green tea–bags	A, a’	b, b’, c’	A, B’	a, A’
	Green tea–loose–spring harvest	D, b’	b, c, c’, d’	E, E’	e, D’
	Green tea–loose–autumn harvest	A, a’	c, d’	A, B’	g, F’
	Correlation-total polyphenol-antioxidant activity	0.9358	0.9324	0.7852	−0.1222
	Correlation-EGCG-antioxidant activity	0.5460	0.6465	0.6113	−0.3081

The same letters A/a/a’/A’ mean no statistically significant differences at the confidence level of *p* ≤ 0.05. A,B,C—effect of temperature 65 °C and 100 °C during 5 min; a,b,c—effect of temperature 65 °C and 100 °C during 10 min; a’,b’,c’—effect of time 5 and 10 min at 100 °C; A’,B’,C’—effect of time 5 and 10 min at 65 °C.

## Data Availability

Data is not reported in any external database.

## Sample Availability

The samples from this batch of tea are no longer available.

## References

[1] Yang C.S., Zhang J., Zhang L., Huang J., Wang Y. (2016). Mechanisms of body weight reduction and metabolic syndrome alleviation by tea. Mol. Nutr. Food Res..

[2] Kumar G., Xu B.J. (2017). A critical review on polyphenols and health benefits of black soybeans. Nutrients.

[3] Yan Z., Zhong Y., Duan Y., Chen Q., Li F. (2020). Antioxidant mechanism of tea polyphenols and its impact on health benefits. Anim. Nutr..

[4] Li Q., Li J., Liu S., Huang J., Lin H., Wang K., Cheng X., Liu Z.A. (2015). Comparative proteomic analysis of the buds and the young expanding leaves of the tea plant (*Camellia sinensis* L.). Int. J. Mol. Sci..

[5] Prasanth M.I., Sivamaruthi B.S., Chaiyasut C., Tencomnao T. (2019). A Review of the Role of Green Tea (*Camellia sinensis*) in Antiphotoaging, Stress Resistance, Neuroprotection, and Autophagy. Nutrients.

[6] Kim Y., Kim M.K. (2019). Effects of different harvesting times and oxidative fermentation methods on phytochemicals, flavors, and sensory properties of Korean teas. Chemistry of Korean Foods and Beverages.

[7] Chan E.C., Tie P.P., Soh E.Y., Law Y.P. (2011). Antioxidant and antibacterial properties of green, black, and herbal teas of *Camellia sinensis*. Pharmacogn. Res..

[8] Chu C., Deng J., Man Y., Qu Y. (2017). Green tea extracts epigallocatechin-3-gallate for different treatments. Biomed. Res. Int..

[9] Koca İ., Bostancı Ş. (2014). Production, composition, and health effects of oolong tea. Turk. J. Agric.-Food Sci. Technol..

[10] Zhao M., Su X.Q., Nian B., Chen L.J., Zhang D.L., Duan S.M., Wang L.Y., Shi X.Y., Jiang B., Jiang W.W. (2019). Integrated meta-omics approaches to understand the microbiome of spontaneous fermentation of traditional Chinese pu-erh tea. Msystems.

[11] Cory H., Passarelli S., Szeto J., Tamez M., Mattei J. (2018). The Role of Polyphenols in human health and food systems: A mini-review. Front. Nutr..

[12] Zeng L., Ma M., Li C., Luo L. (2017). Stability of tea polyphenols solution with different pH at different temperatures. Int. J. Food Prop..

[13] Ioannou I., Chekir L., Ghoul M. (2020). Effect of heat treatment and light exposure on the antioxidant activity of flavonoids. Processes.

[14] Tounekti T., Joubert E., Hernández I., Munné-Bosch S. (2013). Improving the polyphenol content of tea. CRC Crit. Rev. Plant Sci..

[15] Gao G., Chen H., Liu P., Hao Z., Ma G., Chai Y., Wang H., Lu C. (2017). Residue pattern of polycyclic aromatic hydrocarbons during green tea manufacturing and their transfer rates during tea brewing. Food Addit. Contam..

[16] Sanaeifar A., Huang X., Chen M., Zhao Z., Ji Y., Li X., He Y., Zhu Y., Chen X., Yu X. (2020). Nondestructive monitoring of polyphenols and caffeine during green tea processing using Vis-NIR spectroscopy. Food Sci. Nutr..

[17] Lee M.-K., Kim H.-W., Lee S.-H., Kim Y.J., Asamenew G., Choi J., Lee J.-W., Jung H.-A., Yoo S.M., Kim J.-B. (2019). Characterization of catechins, theaflavins, and flavonols by leaf processing step in green and black teas (*Camellia sinensis*) using UPLC-DAD-QToF/MS. Eur. Food Res. Technol..

[18] Hilal Y. (2017). Morphology, manufacturing, types, composition and medicinal properties of tea (*Camellia sinensis*). J. Basic Appl..

[19] Pou K.R.J., Sanjib K.P., Santanu M., Grumezescu A.M., Holban A.M. (2019). Industrial processing of CTC black tea. Caffeinated and Cocoa Based Beverages, Woodhead 8: The Science of Beverages.

[20] Shannon E., Jaiswal A.K., Abu-Ghannam N. (2018). Polyphenolic content and antioxidant capacity of white, green, black, and herbal teas: A kinetic study. Food Res..

[21] Cleverdon R., Elhalaby Y., McAlpine M.D., Gittings W., Ward W.E. (2018). Total Polyphenol Content and Antioxidant Capacity of Tea Bags: Comparison of Black, Green, Red Rooibos, Chamomile and Peppermint over Different Steep Times. Beverages.

[22] Komes D., Horžić D., Belščak A., Ganić K.K., Vulić I. (2010). Green tea preparation and its influence on the content of bioactive compounds. Food Res. Int..

[23] Jakubczyk K., Kochman K., Kwiatkowska A., Kałdunska J., Dec K., Kawczuga D., Janda K. (2020). Antioxidant properties and nutritional composition of Matcha green tea. Foods.

[24] Bae J., Kim N., Shin Y., Kim S.-Y., Kim Y.-J. (2020). Activity of catechins and their applications. Biomed. Dermatol..

[25] Li F., Wang Y., Li D., Chen Y., Qiao X., Fardous R., Lewandowski A., Liu J., Chan T.H., Dou Q.P. (2018). Perspectives on the recent developments with green tea polyphenols in drug discovery. Expert Opin. Drug Discov..

[26] Tang Z., Su Y., Er M.J., Qi F., Zhang L., Zhou J. (2015). A local binary pattern-based texture descriptors for classification of tea leaves. Neurocomputing.

[27] Chacko S.M., Thambi P.T., Kuttan R., Nishigaki I. (2010). Beneficial effects of green tea: A literature review. Chin. Med..

[28] Pou K.R.J. (2016). Fermentation: The key step in the processing of black tea. J. Biosyst. Eng..

[29] Blum-Silva C.H., Chaves V.C., Schenkel E.P., Coelho G.C., Reginatto F.H. (2015). The influence of leaf age on methylxanthines, total phenolic content, and free radical scavenging capacity of Ilex paraguariensis aqueous extracts. Rev. Bras. Farmacogn..

[30] Fujioka K., Iwamoto T., Shima H., Tomaru K., Saito H., Ohtsuka M., Yoshidome A., Kawamura Y., Manome Y. (2016). The powdering process with a set of ceramic mills for green tea promoted catechin extraction and the ROS inhibition effect. Molecules.

[31] ISO (2000). PN-ISO 1026:2000. Fruit and Vegetable–Determination of Dry Matter Content from Drying under Reduced Pressure and Water Content by Azeotropic Distillation.

[32] Musci M., Yao S. (2017). Optimization and validation of Folin–Ciocalteu method for the determination of total polyphenol content of Pu-erh tea. Int. J. Food Sci. Nutr..

[33] Drużynska B., Kostrzewski K., Majewska E., Kowalska J., Derewiaka D., Ciecierska M. (2015). Content of components and their bioactive antioxidant activity sprouts seeds. Zesz. Probl. Postępów Nauk. Rol..

[34] Bienia B., Uram-Dudek A., Dykiel M., Krochmal-Marczak B., Sawicka B. (2019). Antioxidant properties of selected green teas. Herbalism.

[35] Singleton V.L., Rossi J.A. (1965). Colorimetry of total phenolics with phosphomolybdic-phosphotungstic acid reagents. Am. J. Enol. Vitic..

